# Sexual orientation identity disparities in health behaviors, outcomes, and services use among men and women in the United States: a cross-sectional study

**DOI:** 10.1186/s12889-016-3467-1

**Published:** 2016-08-17

**Authors:** Chandra L. Jackson, Madina Agénor, Dayna A. Johnson, S. Bryn Austin, Ichiro Kawachi

**Affiliations:** 1 Clinical and Translational Science Center, Harvard Catalyst, Harvard Medical School, 10 Shattuck Street, Boston, MA 02215 USA; 2 Department of Social and Behavioral Sciences, Harvard T.H. Chan School of Public Health, Boston, MA USA; 3 Center for Community-Based Research, Dana-Farber Cancer Institute, Boston, MA USA; 4 Division of Sleep and Circadian Disorders, Brigham and Women’s Hospital, Harvard Medical School, Boston, MA USA; 5 Division of Adolescent and Young Adult Medicine, Boston Children’s Hospital, Boston, MA USA

**Keywords:** Sexual orientation, Health behaviors, Health outcomes, United States

## Abstract

**Background:**

Research shows that sexual minorities (e.g., lesbian, gay, and bisexual individuals) experience higher levels of discrimination, stigma, and stress and are at higher risk of some poor health outcomes and health behaviors compared to their heterosexual counterparts. However, the majority of studies have examined sexual orientation disparities in a narrow range of health outcomes and behaviors using convenience samples comprised of either men or women living in restricted geographic areas.

**Methods:**

To investigate the relationship between sexual orientation identity and health among U.S. women and men, we used Poisson regression with robust variance to estimate prevalence ratios for health behaviors, outcomes, and services use comparing sexual minorities to heterosexual individuals using 2013 and 2014 National Health Interview Survey data (*N* = 69,270).

**Results:**

Three percent of the sample identified as sexual minorities. Compared to heterosexual women, lesbian (prevalence ratio (PR) = 1.65 [95 % confidence interval (CI): 1.14, 2.37]) and bisexual (PR = 2.16 [1.46, 3.18]) women were more likely to report heavy drinking. Lesbians had a higher prevalence of obesity (PR = 1.20 [1.02, 1.42]), stroke (PR = 1.96 [1.14, 3.39]), and functional limitation (PR = 1.17 [1.02, 1.34] than heterosexual women. Gay men were more likely to have hypertension (PR = 1.21 [1.03, 1.43]) and heart disease (PR = 1.39 [1.02, 1.88]). Despite no difference in health insurance status, sexual minorities were more likely than heterosexual individuals to delay seeking healthcare because of cost; however, members of this group were also  more likely to have received an HIV test and initiated HPV vaccination.

**Conclusion:**

Sexual minorities had a higher prevalence of some poor health behaviors and outcomes.

## Background

The U.S. Department of Health and Human Services has identified understanding and improving the health of lesbian, gay, bisexual, and transgender (LGBT) populations as a national priority. Indeed, Healthy People 2020 has set new objectives and targets for monitoring and promoting LGBT health [1]. Moreover, the Institute of Medicine released a report underscoring the need to conduct additional research on the health of all LGBT populations across the life course, including using national probability samples [2].

Studies show that many sexual minorities – namely, individuals who self-identify as gay, lesbian, or bisexual and who engage in same-gender sexual behavior or report same-gender sexual attractions but do not self-identify as such – have a higher prevalence of health risk behaviors [3, 4] and poor health outcomes [4–8] and a lower prevalence of access to health insurance as well as healthcare [4, 5, 7, 9] compared to their heterosexual counterparts, even after controlling for socioeconomic position (SEP). Few studies, however, have generated nationally representative estimates for a range of health and healthcare measures by sexual orientation identity while using a sample size large enough to provide estimates for both U.S. men and women in the same study.

Therefore, we examined sexual orientation identity disparities in health behaviors, health outcomes, and healthcare access and utilization indicators using data from the 2013 and 2014 National Health Interview Survey (NHIS), which is based on a national probability sample of adult U.S. men and women. We also investigated the relationship between sexual orientation identity and health and healthcare indicators stratified by two age groups. Our study extends the findings of a recent study that used NHIS data to assess sexual orientation identity disparities in health by investigating understudied aspects of sexual minority health related to a broad set of health behaviors, outcomes, and healthcare services and utilization measures [10]. This study also extends previous findings by adjusting for potential confounders and using a larger sample size among both men and women. Based on the minority stress model, which postulates that sexual orientation disparities in health are due to sexual minorities’ unique exposure to stigma and discrimination that can be experienced differently depending on gender and age group [11–15], we hypothesized that sexual minorities have a higher prevalence of health behaviors and poor health outcomes and a lower prevalence of health services use compared to heterosexual persons across gender and age groups. These data will help future studies identify potential drivers of disparities among sexual minorities and test mechanisms posited by the Minority Stress Model.

## Methods

### The national health interview survey

We analyzed data from the National Health Interview Survey (NHIS), which is a series of cross-sectional, nationally representative surveys that use a three-stage stratified cluster probability sampling design to conduct in-person interviews in the households of non-institutionalized U.S. civilians. A detailed description of NHIS procedures has been published elsewhere [16]. Briefly, a probability sample of households was interviewed by trained interviewers from the U.S. Census Bureau to obtain information about health and sociodemographic characteristics of the sampled household on a continuous basis each week. Data were collected using computer-assisted personal interviewing. A randomly selected adult and child (not included in this analysis) provided more specific health-related information. The final response rate for sample adults was 81.7 % for 2013 and was 80.5 % for 2014. The NHIS received written informed consent from each study participant. The Harvard T.H. Chan School of Public Health’s Institutional Review Board approved our study.

### Study participants

Participants included individuals who self-identified as non-Hispanic white, non-Hispanic black/African American, Latino/a or Hispanic, Native American/Alaska Native, and Asian (henceforth, white, black, Latino/a, Native American, Asian) adults aged 18 to 85+ years. Participants were excluded if they had missing data (<3 %) on sexual orientation identity. Our final analytic sample consisted of 69,270 adults.

### Measures

#### Sexual orientation identity

Participants who were asked “Which of the following best represents how you think of yourself?” Response options included: “gay” or “lesbian,” “straight, that is, not lesbian or gay,” “bisexual,” “something else,” and “I don’t know the answer.” The wording of the question differed slightly for men and women, with men being asked if they were “gay” or “straight, that is, not gay” and women being asked if they were “lesbian or gay” or “straight, that is, not lesbian or gay.” Persons who responded “something else” or “I don’t know the answer” were asked follow-up questions about what they meant by “something else” or “don’t know,” but due to sample size constraints and confidentiality, these follow-up responses and potential explanations were not included in the publicly available data.

#### Health behaviors

Leisure-time physical activity was categorized as none, low, or high. Participants engaging in at least some level of activity and providing a specific number of activity bouts were dichotomized at the midpoint and classified as low or high. Participants reporting ‘never’ or ‘unable to do this type activity’ were categorized as ‘none.’ Current smoking status (based on smoking at least 100 cigarettes in entire life) and lifetime alcohol consumption (based on having at least 12 drinks in lifetime and drinking or not in the past year) was categorized as current, former, or never. Based on dietary guidelines, heavy drinking was considered ≥2 drinks per day for men and >1 drink per day for women [17]. We also considered those who consumed five or more drinks on at least 2 days among men and women for 2013 and five or more drinks on at least 2 days among men and four or more drinks on at least 2 days among women for 2014.

Participants reported how many hours of sleep they, on average, obtained in a 24-hour period. Sleep was categorized as <7 h, 7 h, and >7 h. Seven hours of sleep was used as the reference because it has been associated with the lowest levels of morbidity and mortality [18, 19]. As a measure of sadness, participants responded to the question, “During the past 30 days, how often did you feel so sad that nothing could cheer you up?” Participants responded either “all of the time,” “most of the time,” “some of the time,” “a little of the time,” or “none of the time.”

#### Health outcomes

Adults were asked if they had ever been told by a doctor or other health professional that they had “hypertension, also called high blood pressure” and, separately, if they had ever been told they had “diabetes or sugar diabetes,” “cancer,” or a “stroke.” Participants were also asked if a doctor or other health professional ever diagnosed them as having coronary heart disease or any kind of heart condition or disease other than coronary heart disease, angina pectoris, or a myocardial infarction. Self-reported height and weight were used to calculate body mass index (BMI) by dividing measured weight in kilograms by height in meters squared. Obesity was defined as BMI ≥30 kg/m^2^, overweight as 25.0–29.9 kg/m^2^, normal weight as 18.5–24.9 kg/m^2^, and underweight as BMI <18.5 kg/m^2^. Participants were considered to have a functional limitation if they reported being limited in engaging in specific activities because of a physical, mental, or emotional health problem that did not include pregnancy. Participants were considered to have experienced an injury if they reported at least one injury or poisoning episode serious enough to seek medical advice or treatment in the past 3 months.

#### Healthcare access and utilization indicators

Participants reported if they currently had health insurance coverage and whether they had at least one place they usually went when sick or needed health advice. Participants also indicated if they had Medicaid coverage over the past month and the number of times they went to an emergency room regarding their health during the past 12 months, which we categorized as < or ≥2 visits. Participants reported whether, during the past 12 months, they delayed seeking medical care (not including dental care) because of worry about the cost. Men and women aged 18 to 64 years were asked if they ever had an HPV vaccine. Participants were asked if they ever had an HIV test (not including any tests during blood donations). We placed self-reported general health status into three categories (excellent or very good, good, and fair or poor).

#### Sociodemographic characteristics

Participants self-identified with 1 or more of the following categories: white, black/African American, American Indian/Alaskan Native, Asian, and multiple race. Marital status was categorized as married or living with partner, divorced, separated, or widowed, and never married. Educational attainment was categorized as < high school, high school (including general equivalency diploma), some college, and ≥ college-level education. Annual household income was classified as ‘$0–34,999,”35,000–74,999’ and ‘≥$75,000,’ and poverty status was dichotomized for a family or individual income at and above or below the U.S. Census Bureau’s poverty threshold. Based on type of work, we combined occupations into ‘Professional/management,’ ‘Support Services’ and ‘Laborer’ categories. 

### Statistical analysis

We used sampling weights to account for the unequal probabilities of selection resulting from the sample design, survey non-response, and planned oversampling of black, Latino/a, and Asian American individuals and adults aged 65 years and over. Standard errors or variance estimations were calculated using Taylor series linearization. The “subpop” command in Stata, version 13 (Stata Corporation, College Station, Texas, USA) was used for correct variance estimation using the analytic sample. A two-sided *p*-value <0.05 was considered statistically significant.

We used the direct-adjustment method to calculate age-standardized prevalence estimates of sociodemographic characteristics, health behaviors, health outcomes, and healthcare access and utilization indicators among U.S. men and women (separately) by sexual orientation identity. The age distribution from the 2010 U.S. Census was used as the standard population.

Poisson regression with robust error variance was used to estimate gender-specific prevalence ratios for health behaviors, health outcomes, and services use comparing sexual minority and heterosexual individuals [20]. Prevalence ratios were also estimated separately for younger and older age groups. Determined by sample size and distribution, the younger age group included individuals aged 18–30 years, and the older age group was composed of individuals aged 31–85+ years. The aforementioned covariates selected *a priori* as potential confounders included age, race/ethnicity, educational attainment, annual household income, occupational class, health status, and region of residence.

## Results

### Characteristics of the study sample

The final analytic sample consisted of 69,270 participants. Table 1 shows the age-standardized prevalence of sociodemographic characteristics, health behaviors, health outcomes, and health services use by sexual orientation identity among U.S. men and women. Participants’ mean age was 49.9 ± 0.07 years; 52 % were women, and 3 % identified as sexual minorities. Gay and bisexual men and lesbians were generally more likely than heterosexual individuals to have at least a college education. Participants who reported not knowing their sexual orientation identity or responded “don’t know or something else” were more likely than heterosexual individuals to live in poverty and be in fair or poor general health. Many bisexual men had at least a college education (46 %) and some lived in poverty (15 %). In comparison, fewer heterosexual men had a college education (30 %), but fewer heterosexual men live in poverty (11 %).

**Table 1:** Age-standardized Socio-demographic Characteristics, Health Behaviors, and Health Outcomes by Sexual Orientation Identity among U.S. Men and Women, National Health Interview Survey, 2013 and 2014 (*N* = 69,270)

	Men (*n* = 30,961)								Women (*n* = 38,309)							
	Heterosexual		Gay		Bisexual		“Something else”/Do not know		Heterosexual		Lesbian		Bisexual		“Something else”/Do not know	
	n	%	n	%	n	%	n	%	n	%	n	%	n	%	n	%
Sample size	29,967	97.2	624	1.8	162	0.4	208	0.5	37,185	97.2	525	1.4	353	0.9	246	0.5
Sociodemographics																
Race/ethnicity																
White	18,848	70.1	413	76.2	105	73.1	96	52.7	22,542	68.3	315	71.4	231	73.5	123	56.4
Black	3,918	10.7	79	9.8	19	7.6	34	14.6	5,699	12.3	100	12.7	61	16.0	38	11.0
Latino/Hispanic	4,787	13.0	96	10.8	21	7.8	43	19.8	6,086	12.9	77	12.5	43	7.2	51	17.5
Native American	398	1.0	5	0.4	2	1.1	5	1.5	446	0.9	9	1.1	9	2.6	7	1.3
Asian/Pacific Islander	1,873	5.2	28	2.8	15	10.4	29	11.4	2,235	5.6	18	2.3	4	0.7	25	13.8
Educational attainment																
< High school	8,832	29.4	130	19.9	28	18.0	53	29.0	10,182	28.2	116	22.8	84	21.0	69	25.4
High school graduate	3,831	11.8	24	5.8	14	6.8	47	22.7	4,826	11.1	31	3.9	41	12.7	65	25.8
Some college	8,651	28.5	197	33.1	63	28.9	44	24.1	11,776	31.6	190	34.9	122	37.3	54	25.6
≥ College	8,534	30.3	273	41.2	57	46.3	58	24.2	10,242	29.1	187	38.4	106	29.0	53	23.2
Marital status																
Married	14,501	61.1	78	19.2	31	22.0	38	20.6	15,370	52.7	89	23.6	60	30.8	53	30.8
Divorced/separated/widowed	6,652	17.3	71	16.1	35	26.6	52	24.8	12,741	27.6	103	22.2	95	32.9	77	27.6
Never married	8,761	21.6	472	64.7	96	51.4	116	54.6	8,972	19.7	329	54.2	194	36.3	116	41.6
Unemployed (yes)	19,407	37.5	438	40.9	98	52.2	107	49.8	19,634	47.3	344	43.1	215	49.2	124	51.1
Annual Household income (<$35,000 per year)	10,769	29.3	229	31.9	78	36.0	120	43.3	15,607	34.7	215	32.7	192	45.4	147	53.7
Living in poverty (<100 % Federal Poverty Level)	4,284	11.1	79	10.4	33	15.2	70	29.4	6,885	14.1	102	12.3	105	23.3	73	25.9
Occupation																
Professional/management	6,339	74.0	191	75.4	38	77.9	26	74.3	5,213	73.1	110	72.9	53	70.1	20	74.4
Support Services	7,262	15.6	279	12.5	61	11.0	49	8.9	20,892	20.3	264	18.3	199	16.9	110	21.6
Laborers	14,578	10.4	140	12.1	53	11.1	110	16.8	7,615	6.6	120	8.8	79	13.0	68	4.0
Region of residence																
Northeast	4,797	18.0	114	19.6	24	16.1	36	16.3	6,036	17.7	103	22.9	52	13.8	35	16.7
Midwest	6,392	23.1	92	13.9	28	22.3	40	18.4	7,654	22.4	92	19.0	70	27.6	45	17.6
South	10,528	36.5	221	35.7	47	32.5	63	33.3	13,754	38.0	189	33.5	120	32.5	85	38.4
West	8,250	22.4	197	30.8	63	29.1	69	32.0	9,741	21.9	141	24.6	111	26.1	81	27.3
Health behaviors																
Sleep duration																
< 7 h	9,157	30.6	198	30.5	52	25.8	60	30.2	11,570	30.7	190	33.6	123	29.6	74	32.5
7 h	8,835	29.7	190	30.4	53	36.4	61	32.6	10,062	28.1	140	27.4	86	32.9	47	17.4
> 7 h	11,708	39.7	232	39.1	57	37.8	80	37.2	15,155	41.2	188	39.0	139	37.5	111	50.1
Smoking status																
Never	15,919	52.1	307	48.1	87	50.4	110	51.8	24,306	64.8	265	50.5	178	51.7	164	73.1
Former	7,941	29.2	152	30.2	32	23.4	46	21.6	7,166	20.6	113	28.2	73	32.4	45	16.5
Current	6,067	18.7	164	21.7	43	26.2	51	26.6	5,666	14.6	147	21.3	102	15.9	37	10.4
Alcohol consumption																
Never	4,402	14.7	46	6.4	20	9.5	41	19.9	9,874	26.0	74	14.2	51	24.1	92	42.6
Current	20,545	69.0	516	81.1	126	80.1	128	60.0	21,360	58.7	381	67.2	274	64.7	115	41.3
Former	4,722	16.3	59	12.5	16	10.4	38	20.1	5,674	15.3	67	18.6	28	11.2	37	16.1
Heavy drinking ^a^	1,761	6.8	42	6.5	15	12.9	15	6.1	1,785	5.7	41	9.7	46	15.6	18	6.3
5+ drinks on at least 2 days ^b^	7,538	32.2	188	29.7	61	45.0	43	30.5	4,357	17.5	117	24.1	107	31.7	33	19.1
Leisure-time physical activity																
Never/unable	9,010	30.7	129	22.1	41	25.7	64	32.8	12,858	34.3	158	32.1	69	18.1	89	31.8
Low	9,660	32.8	209	34.4	53	35.5	67	29.8	11,568	31.6	174	35.0	120	35.0	75	35.7
High	11,169	36.5	285	43.5	68	38.8	76	37.4	12,669	34.1	190	32.9	161	46.9	80	32.5
Sad (past 30 days) (≥mostly)	850	2.7	28	3.4	3	2.4	11	4.8	1,487	3.7	32	5.6	31	6.8	15	8.0
Health outcomes																
Overweight prevalence ^c^	20,868	72.1	372	63.8	91	48.4	117	61.3	20,885	58.5	325	63.8	199	64.3	132	57.1
Obesity prevalence ^d^	8,506	29.8	145	25.0	45	19.1	44	18.8	10,724	29.4	183	36.7	123	38.3	72	33.1
Hypertension (yes)	9,915	37.0	189	39.0	40	32.2	73	34.4	12,491	35.5	134	32.2	68	32.1	82	36.5
Diabetes (yes)	3,183	12.5	54	10.2	13	11.4	26	10.4	3,857	10.7	38	7.7	17	7.1	33	16.2
Cancer (yes)	2,471	10.2	58	13.4	10	12.7	10	4.2	3,718	10.8	46	11.0	31	16.0	13	4.4
Heart disease (yes)	3,543	13.5	75	15.2	18	16.9	27	15.6	3,905	10.8	44	9.9	26	7.2	27	11.1
Stroke (yes)	937	3.5	10	1.9	3	1.1	11	8.2	1,198	3.2	27	5.8	7	3.4	10	2.8
Functional limitation (yes)	9,481	34.8	181	35.4	56	37.7	95	52.0	15,472	43.1	226	49.6	147	46.8	125	53.2
Any Injury (3 months)	970	3.0	29	3.5	6	1.5	6	2.2	1,264	2.9	26	4.9	33	6.5	11	2.6
Healthcare access and utilization																
Health insurance (no)	5,096	14.0	96	12.4	26	17.8	52	24.5	4,982	12.0	80	12.0	69	11.9	40	15.4
Medicaid (yes)	2,081	6.1	47	7.5	13	7.6	35	14.6	4,490	9.5	77	10.9	59	18.3	42	14.5
Usual place of care (yes)	23,695	84.2	505	87.3	121	82.5	142	72.5	32,937	90.9	432	87.4	283	89.7	197	85.5
# ER visits in past year ≥2	1,689	31.7	36	36.4	13	37.1	19	49.0	3,153	38.8	60	37.1	58	52.6	30	44.1
Delayed healthcare because of cost (yes)	2,986	8.2	99	10.8	33	20.5	31	12.7	4,288	10.5	105	15.6	86	13.1	39	10.8
HPV vaccine (initiation, yes)	535	2.0	31	4.8	8	4.5	5	3.7	2,756	8.6	59	10.1	85	18.2	15	5.0
HIV testing (ever, yes)	10,257	32.0	525	79.0	95	62.5	83	38.5	14,591	36.0	268	48.7	222	52.1	93	29.9
Health status																
Excellent/very good	17,795	58.2	397	62.7	94	59.2	95	41.9	21,130	57.4	298	55.1	199	47.6	111	47.5
Good	7,940	27.2	149	23.7	43	20.5	66	30.7	10,338	27.5	138	24.6	92	39.0	81	34.3
Fair/poor	4,223	14.6	78	13.6	25	20.3	47	27.4	5,694	15.1	89	20.3	62	13.4	51	18.2

Note. All estimates are weighted for the survey’s complex sampling design

*ER* emergency room; ^a^ heavy drinking= > 2 drinks per day for men and >1 drink per day for women; ^b^ 5+ drinks on at least 2 days among men and women in 2013 and 4+ drinks on at least 2 days among women in 2014 only; ^c^ Overweight = ≥25 kg/m^2^; ^d^ Obesity = ≥30 kg/m^2^; *HPV* human papillomavirus, *HIV* human immunodeficiency virus

### Association between sexual orientation identity and health behaviors

Adjusting prevalence ratios for potential confounders, heavy drinking ranged from 65 % (prevalence ratio (PR) = 1.65 [95 % confidence interval (CI): 1.14, 2.37]) higher for lesbians to an over 2-fold higher prevalence (PR = 2.16 [1.46, 3.18]) among bisexual women compared to their heterosexual counterparts (Table 2). Bisexual men and women were also more likely than heterosexuals to report consuming ≥5 drinks on at least 2 days in the past year. Bisexual women and men and those who responded “don’t know or something else” were less likely to report never engaging or being unable to engage in physical activity. In contrast, there were no differences in sleep duration by sexual orientation identity among men and women.

**Table 2:** Fully-Adjusted Prevalence Ratios for Health Behaviors and Healthcare Access and Utilization Indicators in Relation to Sexual Orientation Identity among U.S. Men and Women, National Health Interview Survey, 2013 and 2014 (*N* = 69,270)

	Men (*n* = 30,961) (reference: heterosexual)			Women (*n* = 38,309) (reference: heterosexual)		
	Gay PR (95 % CI)	Bisexual PR (95 % CI)	“Something else”/Do not know PR (95 % CI)	Lesbian PR (95 % CI)	Bisexual PR (95 % CI)	“Something else”/Do not know PR (95 % CI)
Sample size	*n* = 624	*n* = 162	*n* = 208	*n* = 525	*n* = 353	*n* = 246
Health Behavior						
Alcohol consumption (reference: never)						
Current	**1.09 (1.06–1.13)**	**1.09 (1.03–1.16)**	0.94 (0.80–1.11)	**1.14 (1.08–1.20)**	1.03 (0.93–1.14)	0.86 (0.70–1.06)
Former	1.23 (0.99–1.53)	1.07 (0.71–1.61)	0.88 (0.63–1.23)	**1.30 (1.05–1.60)**	0.65 (0.37–1.16)	0.88 (0.57–1.35)
Heavy drinking^a^	0.89 (0.58–1.38)	1.81 (0.98–3.33)	0.95 (0.44–2.02)	**1.65 (1.14–2.37)**	**2.16 (1.46–3.18)**	**1.92 (1.08–3.39)**
5+ drinks on at least 2 days^b^	0.97 (0.82–1.15)	**1.29 (1.05–1.57)**	0.95 (0.65–1.38)	1.22 (0.98–1.52)	**1.34 (1.08–1.66)**	1.37 (0.94–1.98)
Leisure-time physical activity (reference: high)						
Low	0.89 (0.76–1.04)	0.99 (0.75–1.29)	0.88 (0.66–1.18)	1.05 (0.91–1.20)	0.92 (0.75–1.13)	0.97 (0.75–1.26)
Never/Unable	0.89 (0.73–1.09)	0.94 (0.68–1.32)	0.78 (0.59–1.05)	1.01 (0.85–1.19)	**0.56 (0.42–0.75)**	**0.74 (0.55–0.99)**
Smoking status (reference: never)						
Current	**1.39 (1.14–1.70)**	1.17 (0.91–1.51)	1.32 (0.95–1.82)	**1.47 (1.21–1.80)**	1.26 (0.98–1.61)	0.87 (0.54–1.40)
Former	1.11 (0.94–1.32)	0.90 (0.94–1.32)	0.85 (0.62–1.17)	**1.49 (1.22–1.82)**	**1.43 (1.09–1.86)**	0.82 (0.56–1.20)
Sleep duration (reference: 7 h)						
<7 h	1.09 (0.95–1.24)	0.78 (0.50–1.20)	0.93 (0.63–1.36)	1.00 (0.86–1.15)	1.02 (0.86–1.22)	1.20 (0.97–1.48)
>7 h	1.08 (0.96–1.22)	0.95 (0.74–1.23)	0.99 (0.80–1.21)	0.87 (0.75–1.01)	1.04 (0.89–1.22)	1.16 (0.99–1.36)
Healthcare access and utilization						
Health insurance (no)	1.12 (0.86–1.45)	1.05 (0.68–1.62)	1.24 (0.82–1.62)	1.05 (0.80–1.38)	1.09 (0.81–1.48)	1.12 (0.66–1.90)
Usual place for care (yes)	**1.06 (1.01–1.11)**	1.04 (0.94–1.14)	0.94 (0.82–1.08)	0.94 (0.90–0.99)	0.92 (0.84–1.01)	0.96 (0.89–1.05)
ER visits (≥2 in past year)	1.04 (0.71–1.52)	1.28 (0.69–2.38)	1.36 (0.82–2.24)	1.01 (0.79–1.30)	0.92 (0.69–1.22)	1.22 (0.83–1.79)
Delay healthcare because of costs (yes)	**1.40 (1.08–1.81)**	**2.00 (1.44–2.79)**	0.98 (0.57–1.68)	**1.47 (1.15–1.88)**	**1.65 (1.28–2.13)**	0.95 (0.60–1.51)
HPV vaccine (initiation)	**2.45 (1.53–3.92)**	**2.31 (1.00–5.35)**	1.77 (0.67–4.68)	1.10 (0.81–1.50)	**1.46 (1.16–1.84)**	0.55 (0.26–1.17)
HIV test (ever)	**2.25 (2.12–2.40)**	**1.67 (1.41–1.97)**	1.24 (0.97–1.58)	**1.22 (1.09–1.36)**	**1.17 (1.02–1.34)**	0.82 (0.62–1.07)

*PR* prevalence ratio, *CI* confidence interval; ^a^heavy drinking= > 2 drinks per day for men and >1 drink per day for women; ^b^5+ drinks on at least 2 days among men and women in 2013 and 4+ drinks on at least 2 days among women in 2014 only; *h* hours, *ER* emergency room, *HPV* human papillomavirus, *HIV* human immunodeficiency virus. Adjusted for age, race/ethnicity, educational attainment, income, occupational class, health status, and region of residence. Note. All estimates are weighted for the survey’s complex sampling design. Boldface indicates statistically significant results at the 0.05 level

### Association between sexual orientation identity and health outcomes

Compared to heterosexual women, lesbians had a 20 % higher prevalence of obesity PR = 1.20 [1.02, 1.42]), 96 % higher prevalence of stroke (PR = 1.96 [1.14, 3.39]), and 17 % higher prevalence of having a functional limitation (PR = 1.17 [1.02, 1.34]) (Table 3). Bisexual women had over a two-fold higher prevalence of sustaining an injury or poisoning in the past 3 months (PR = 2.49 [1.44, 4.32]) and of mostly feeling so sad that nothing could cheer them up during the past 30 days (PR = 2.10 [1.25, 3.54]). Gay men were 21 % (PR = 1.21 [1.03, 1.43]) more likely to have hypertension and 39 % (PR = 1.39 [1.02, 1.88]) more likely to have heart disease compared to heterosexual men. Similarly, sexual minority men were more likely than heterosexual men to have a functional limitation.

**Table 3:** Fully-adjusted Prevalence Ratios for Health Outcomes in Relation to Sexual Orientation Identity Disparities among U.S. Men and Women, National Health Interview Survey, 2013 and 2014 (*N* = 69,270)

	Men (*n* = 30,961) (reference: heterosexual)			Women (*n* = 38,309) (reference: heterosexual)		
	Gay PR (95 % CI)	Bisexual PR (95 % CI)	“Something else”/Do not know PR (95 % CI)	Lesbian PR (95 % CI)	Bisexual PR (95 % CI)	“Something else”/Do not know PR (95 % CI)
	*n* = 624	*n* = 162	*n* = 208	*n* = 525	*n* = 353	*n* = 246
Sad (past 30 days) (≥mostly)	1.73 (0.98–3.06)	1.58 (0.98–3.06)	1.54 (0.60–3.99)	1.31 (0.82–2.08)	**2.10 (1.25–3.54)**	2.53 (0.97–6.63)
Hypertension (yes)	**1.21 (1.03–1.43)**	0.83 (0.57–1.22)	0.76 (0.57–1.03)	0.91 (0.74–1.12)	0.96 (0.71–1.31)	0.98 (0.75–1.28)
Overweight ^a^ (yes)	**0.85 (0.77–0.93)**	**0.81 (0.65–1.00)**	0.85 (0.71–1.01)	1.09 (0.98–1.22)	1.07 (0.93–1.22)	0.96 (0.80–1.15)
Obesity ^b^ (yes)	0.90 (0.74–1.09)	0.97 (0.69–1.37)	**0.56 (0.38–0.83)**	**1.20 (1.02–1.42)**	1.16 (0.93–1.45)	1.06 (0.79–1.42)
Diabetes (yes)	0.83 (0.59–1.16)	0.93 (0.47–1.84)	0.58 (0.33–1.05)	0.88 (0.58–1.34)	0.63 (0.33–1.20)	1.31 (0.82–2.11)
Cancer (yes)	1.32 (0.95–1.83)	1.23 (0.48–3.10)	**0.33 (0.14–0.80)**	1.13 (0.78–1.63)	**1.94 (1.18–3.17)**	**0.34 (0.16–0.71)**
Heart disease (yes)	**1.39 (1.02–1.88)**	1.38 (0.79–2.43)	1.03 (0.60–1.78)	0.91 (0.61–1.35)	0.73 (0.40–1.35)	0.68 (0.37–1.24)
Stroke (yes)	0.76 (0.34–1.70)	**0.04 (0.005–0.26)**	1.33 (0.54–3.30)	**1.96 (1.14–3.39)**	1.68 (0.71–3.97)	0.45 (0.17–1.21)
Any functional limitation (yes)	**1.24 (1.05–1.46)**	**1.30 (1.00–1.70)**	**1.35 (1.10–1.66)**	**1.17 (1.02–1.34)**	**1.41 (1.15–1.73)**	1.14 (0.88–1.47)
Any Injury (past 3 months) (yes)	0.96 (0.59–1.56)	0.81 (0.29–2.31)	0.44 (0.14–1.41)	1.84 (0.95–3.57)	**2.49 (1.44–4.32)**	1.10 (0.44–2.76)

Note. All estimates are weighted for the survey’s complex sampling design and adjusted for age (18–30, 31–49, 50–64, 65+) race/ethnicity, educational attainment, income, occupational class, health status, and region of residence; Boldface indicates statistically significant results at the 0.05 level

*PR* prevalence ratio, *CI* confidence interval. ^a^ Overweight = ≥25 kg/m^2^; ^b^ Obesity = ≥30 kg/m^2^

### Association between sexual orientation identity and healthcare access and utilization

Despite no difference in health insurance status and emergency room visits, gay men (PR = 1.40 [1.08, 1.81]) and lesbians (PR = 1.47 [1.15, 1.88]) as well as bisexual men (PR = 2.00 [1.44, 2.79]) and women (PR = 1.65 [1.28, 2.13]) were more likely than their heterosexual counterparts to delay seeking healthcare because of cost. In contrast, these groups were more likely to have initiated HPV vaccination and received an HIV test. Gay (PR = 1.06 [1.01, 1.11]) but not bisexual (PR = 1.04 [0.94, 1.14]) men were more likely to have a usual place for care compared to heterosexual men.

### Age differences in health behaviors and outcomes by sexual orientation identity

Table 4 shows fully adjusted prevalence ratios for health behaviors and healthcare access and utilization indicators in relation to sexual orientation identity among men and women, stratified by age group (aged 18–30 years and 31–85+ years) based on sample size. Compared to their heterosexual counterparts, younger and older gay men and lesbians were more likely to report being current drinkers. Younger bisexual women were 44 % (PR = 1.44 [1.01, 2.05]) more likely to be current alcohol consumers compared to their heterosexual counterparts. Older lesbian and bisexual women were more likely than older heterosexual women to be former drinkers and former cigarette smokers. Younger lesbian and bisexual women and older bisexual women were more likely than their heterosexual counterparts to report heavy drinking. Younger (PR = 1.68 [1.09, 2.61]) and older (PR = 1.25 [1.04, 1.52]) gay men were more likely than their heterosexual counterparts to be current smokers.

**Table 4:** Fully-Adjusted Prevalence Ratios of Sexual Orientation Disparites in Health Behaviors and Healthcare Access and Utilization Indicators among 69,270 U.S. Men and Women by Younger and Older Age Group, National Health Interview Survey, 2013 and 2014

	Men (*n* = 30,961) (reference: heterosexual)				Women (*n* = 38,309) (reference: heterosexual)			
	Gay PR (95 % CI)		Bisexual PR (95 % CI)		Lesbian PR (95 % CI)		Bisexual PR (95 % CI)	
	*n* = 624		*n* = 162		*n* = 525		*n* = 353	
	Younger, PR (95 % CI)	Older, PR (95 % CI)	Younger, PR (95 % CI)	Older, PR (95 % CI)	Younger, PR (95 % CI)	Older, PR (95 % CI)	Younger, PR (95 % CI)	Older, PR (95 % CI)
	*n* = 147	*n* = 477	*n* = 59	*n* = 103	*n* = 143	*n* = 382	*n* = 172	*n* = 181
Health behavior								
Alcohol consumption (reference: never)								
Current	**1.68 (1.09–2.61)**	**1.25 (1.04–1.52)**	1.27 (0.85–1.88)	1.14 (0.83–1.57)	**1.51 (1.08–2.10)**	**1.47 (1.16–1.87)**	**1.44 (1.01–2.05)**	1.18 (0.85–1.64)
Former	0.90 (0.49–1.66)	1.13 (0.95–1.35)	0.54 (0.16–1.88)	0.99 (0.62–1.56)	0.83 (0.29–2.33)	**1.53 (1.25–1.88)**	1.24 (0.68–2.25)	**1.57 (1.18–2.08** **)**
Heavy drinking^a^	0.72 (0.32–1.59)	0.96 (0.58–1.59)	2.01 (0.86–4.66)	1.63 (0.66–3.99)	**2.69 (1.57–4.62)**	1.29 (0.80–2.07)	**1.81 (1.01–3.22)**	**2.61 (1.60–4.24)**
5+ drinks on at least 2 days^b^	1.00 (0.76–1.31)	0.95 (0.77–1.18)	**1.42 (1.19–1.69)**	1.14 (0.78–1.68)	1.11 (0.79–1.56)	**1.34 (1.01–1.77)**	1.13 (0.86–1.49)	**1.75 (1.29–2.37)**
Leisure-time physical activity (reference: high)								
Low	0.68 (0.46–1.03)	0.96 (0.82–1.13)	0.76 (0.46–1.26)	1.11 (0.82–1.51)	1.07 (0.83–1.39)	1.04 (0.89–1.22)	0.93 (0.68–1.25)	0.92 (0.71–1.19)
Never/unable	0.88 (0.52–1.51)	0.89 (0.73–1.09)	1.03 (0.50–2.11)	0.93 (0.66–1.30)	0.96 (0.68–1.36)	1.01 (0.83–1.22)	0.63 (0.39–1.00)	0.51 (0.36–0.72)
Smoking status (reference: never)								
Current	**1.68 (1.09–2.61)**	**1.25 (1.04–1.52)**	1.27 (0.85–2.61)	1.14 (0.83–1.52)	**1.51 (1.08–2.10)**	**1.47 (1.16–1.87)**	**1.88 (1.08–3.27)**	1.18 (0.85–1.87)
Former	0.90 (0.49–1.66)	1.13 (0.95–1.35)	0.54 (0.16–1.88)	0.99 (0.62–1.56)	0.83 (0.29–2.33)	**1.53 (1.25–1.88)**	1.32 (0.60–2.91)	**1.57 (1.18–2.08)**
Sleep duration (reference: 7 h)								
<7 h	1.11 (0.83–1.50)	1.07 (0.93–1.24)	1.07 (0.83–1.50)	0.93 (0.65–1.32)	0.83 (0.61–1.13)	1.05 (0.89–1.23)	1.06 (0.82–1.37)	1.01 (0.79–1.28)
>7 h	1.08 (0.86–1.37)	1.08 (0.94–1.24)	0.95 (0.65–1.38)	0.96 (0.67–1.36)	0.88 (0.69–1.12)	0.88 (0.73–1.06)	0.96 (0.73–1.25)	1.14 (0.95–1.35)
Healthcare access and utilization indicator								
Health insurance (no)	0.90 (0.61–1.33)	1.18 (0.85–1.63)	0.68 (0.30–1.54)	1.51 (0.94–2.42)	1.20 (0.84–1.72)	1.00 (0.66–1.53)	1.26 (0.82–1.93)	1.07 (0.75–1.54)
Usual healthcare place (yes)	**1.13 (1.01–1.27)**	1.04 (0.99–1.09)	1.09 (0.82–1.45)	**1.23 (1.01–1.50)**	0.87 (0.75–1.02)	0.96 (0.92–1.01)	0.88 (0.75–1.05)	0.96 (0.88–1.04)
ER visits (≥2 in past year)	0.63 (0.29–1.37)	1.23 (0.82–1.86)	1.40 (0.57–3.41)	1.43 (0.68–3.04)	1.08 (0.76–1.53)	0.97 (0.69–1.36)	0.85 (0.57–1.26)	1.16 (0.82–1.63)
Delay healthcare because of costs (yes)	1.44 (0.78–2.67)	**1.40 (1.04–1.89)**	**3.94 (2.10–7.39)**	**1.91 (1.27–2.89)**	**1.65 (1.11–2.44)**	**1.46 (1.07–1.99)**	**2.25 (1.59–3.21)**	1.25 (0.87–1.78)
HPV shot/vaccine (initiation)	1.77 (0.93–3.35)	**4.58 (2.32–9.04)**	**2.55 (1.05–6.20)**	1.76 (0.25–12.38)	1.01 (0.73–1.40)	1.69 (0.80–3.55)	**1.40 (1.10–1.80)**	**2.07 (1.02–4.21)**
HIV test (ever)	**2.24 (1.91–2.62)**	**2.23 (2.10–2.36)**	**1.76 (1.15–2.69)**	**1.67 (1.33–2.09)**	1.05 (0.85–1.29)	**1.27 (1.11–1.45)**	1.14 (0.92–1.41)	**1.32 (1.14–1.53)**

Note. Sample size for the ‘Something else/Don’t know’ sexual minority group was too small for robust statistical estimation. All estimates are weighted for the survey’s complex sampling design and adjusted for age, race/ethnicity, educational attainment, income, occupational class, health status, and region of residence; Boldface indicates statistically significant results at the 0.05 level

*PR* prevalence ratio, *CI* confidence interval; younger adults were aged 18–30 years; older adults were aged 31–85+ years; ^a^ heavy drinking ≥2 drinks per day for men and >1 drink per day for women; ^b^ 5+ drinks on at least 2 days among men and women in 2013 and 4+ drinks on at least 2 days among women in 2014 only; *ER* emergency room, *HPV* human papillomavirus, *HIV* human immunodeficiency virus

While there were no statistically significant sexual orientation differences in health insurance status across age groups, all sexual minorities (with the exception of younger gay men) were more likely than their heterosexual counterparts to delay healthcare due to cost concerns. There were also no sexual orientation differences in emergency room visits across age groups. Both younger (PR = 2.24 [1.91, 2.62]) and older (PR = 2.23 [2.10, 2.36]) gay men as well as younger (PR = 1.76 [1.15, 2.69]) and older (PR = 1.67 [1.33, 2.09]) bisexual men were more likely to report ever getting tested for HIV compared to heterosexual men. Relative to their heterosexual counterparts, older gay (PR = 4.58 [2.32, 9.04]) and younger (PR = 2.55 [1.05, 6.20]) bisexual men as well as younger (PR = 1.40 [1.10, 1.80]) and older (PR = 2.07 [1.02, 4.21]) bisexual women were more likely to initiate HPV vaccination. Only older lesbian (PR = 1.27 [1.11, 1.45]) and bisexual (PR = 1.32 [1.14, 1.53]) women were more likely to have ever been tested for HIV relative to their heterosexual counterparts.

## Discussion

We identified important disparities in health behaviors, health outcomes, and healthcare access and utilization indicators between sexual minorities and heterosexual individuals in a nationally representative sample of U.S. men and women; several of these disparities varied by specific sexual orientation identity, gender, and age groups. We found that heavy drinking ranged from 65 % to an over two-fold higher prevalence among sexual minorities compared to heterosexual individuals. Lesbians were more likely to be obese than heterosexual women, to have suffered a stroke, and to have a functional limitation. Bisexual women had over a two-fold higher prevalence of sustaining an injury/poisoning in the past 3 months, which may be related to alcohol abuse. Potentially related to preventable factors like increased stress or HIV, gay men were more likely to have hypertension and heart disease. Sexual minority men were more likely to have a functional limitation. Regarding age-group differences, younger bisexual women were 44 % more likely to be current alcohol consumers, and older lesbian and bisexual women were more likely than heterosexual women to be former drinkers and former cigarette smokers.

Our study appears fairly consistent with prior studies. For instance, prior research has found that alcohol, tobacco, and other drug use (as well as the morbidities associated with these exposures) were higher among sexual minorities than heterosexuals [21–26]. These studies show that lesbians are more likely to be in recovery and to have been in treatment for alcohol use related problems compared to heterosexual women [25]. High rates of risk factors for heavy drinking and drinking problems – namely, childhood sexual abuse, depression, and suicidal ideation – among lesbians in other studies may explain the sexual orientation disparities in alcohol use that we observed in our analysis [25]. Further, a meta-analysis found that sexual minorities across North America and Europe were much more likely than heterosexual individuals to experience anxiety, depression, and suicidal ideation [27] as well as panic attacks and psychological distress [28], which may explain higher rates of alcohol use and cigarette smoking among sexual minority women, possibly to cope with stigma, discrimination, and stress. Lastly, it is possible that higher rates of obesity among sexual minority women may be due to a higher prevalence of binge eating in this population relative to heterosexual women, which has been linked to minority stress [29].

In this study, older gay men, younger bisexual men, and bisexual women were more likely than their heterosexual counterparts to report initiating HPV vaccination. Agénor et al. found that lesbians aged 15–25 years were less likely to have initiated HPV vaccination relative to their heterosexual counterparts [30]. The difference in these study findings may be due to differences in study population age. Although all sexual minorities (except younger gay men) were more likely than heterosexual persons to delay healthcare because of cost, we observed no sexual orientation identity disparities in health insurance status. In contrast, prior studies have found that sexual minorities were less likely to have health insurance, have received a checkup within in the past year, and have unmet medical needs compared to their heterosexual counterparts [9, 31]. A different study found results similar to ours for women but showed that healthcare access among men in same-sex relationships was the same as or greater than among men in opposite-sex relationships [32]. However, this study used a different dimension of sexual orientation (i.e., gender of sexual partners vs. sexual orientation identity) than our study, which may explain the disparate findings. The demographics of the participants in each study could also contribute to disparate findings. For instance, both affluent and impoverished sexual minorities are included in our nationally representative sample and other studies may have had disproportionate representation from either group. Furthermore, although our study did not identify any difference in sleep duration between sexual minority and heterosexual individuals, prior research has found evidence of shorter sleep duration among sexual minorities compared to their heterosexual counterparts [33].

According to the minority stress model [34], experiences of stigma, discrimination, and victimization related to being a sexual minority may lead to a stress response that increases the risk of poor mental and physical health outcomes among sexual minorities. Specific parts of the stress process linked to being a sexual minority are believed to include expectations of rejection, sexual orientation identity masking, internalized homophobia, and coping by, for instance, attempting to decrease minority stress through problem solving, expressing emotions, and using substances to cope [35]. Discrimination may be a particularly important mechanism by which inequities related to being a sexual minority in U.S. society are related to suboptimal health among sexual minorities. For example, a prior study found that gay and bisexual individuals reported more experiences of lifetime and day-to-day discrimination than heterosexual individuals, and approximately 42 % of sexual minorities attributed this, at least in part, to their sexual orientation. Experiencing discrimination was positively associated with poor quality of life and psychiatric morbidity in this study [36]. Further, interpersonal and institutional discrimination based on sexual orientation identity also contributes to a lack of supportive social and health services for sexual minorities [37] and may help explain sexual orientation identity disparities in healthcare access and utilization among U.S. women and men. Further investigations of the influence of social contexts or norms/mainstream culture on health disparities among sexual minorities are needed.

This study has several limitations. First, we used data from a cross-sectional survey that included only one dimension of sexual orientation (i.e., sexual orientation identity), although the relationship between sexual orientation and health may differ based on the measure of sexual orientation (e.g., sexual attraction, sex of sexual partners) used. Sexuality is fluid and sexual orientation categories are historically contingent. It is possible for participants to shift, for example, from the “something else/don’t know” category to another category over time. Therefore, we need longitudinal studies to examine sexual orientation disparities over the lifecourse and historical time [38, 39]. Second, all data are based on self-report, and some estimates (e.g. BMI), therefore, may be conservative. Third, data on social stressors (e.g., stigma, discrimination) or stress responses were not available for analysis in our study, although they are likely to partially, if not fully, mediate the relationship between sexual orientation identity and many of the health and healthcare outcomes that we considered in this study. Pap smear testing among women was also unavailable. Fourth, there could also be differences in reporting of health and healthcare experiences by sexual orientation identity, which could bias our results; however, such differences have not been documented. Also, the two categories we used to investigate age or cohort differences were broad due to sample size limitations, but it is important to acknowledge that developmental and generational differences exist especially among those aged 31 through older than 85 years.

This study also has important strengths. Specifically, our analyses used data from a large national probability sample of the U.S. population with very little missing data. Therefore, we were able to robustly stratify estimates of health and healthcare by sexual orientation identity by both gender and age, which will help inform future studies and evidence-based interventions that are tailored to the unique needs of sexual minorities with different social, developmental, and health and healthcare needs. We also present findings on understudied aspects of gay and bisexual men’s health as well as results pertaining to the health and healthcare of lesbian and bisexual women, for whom nationally representative data are scarce [40].

## Conclusions

In conclusion, sexual minorities had a higher prevalence of some poor health behaviors and outcomes. In order to help inform evidence-based programs and policies that promote the health and healthcare of sexual minorities, future research identifying the factors that may mediate the relationship between sexual orientation identity and health behaviors, outcomes, and services use is needed – including among U.S. women and men separately given the complex interplay between sexual orientation and gender [41, 42]. Additionally, the interaction between other social factors (e.g., race/ethnicity, SEP) and sexual orientation identity should be investigated in order to inform future health interventions that meet the needs of diverse groups of sexual minorities – including those of color and those from low-income backgrounds, who remain understudied and underserved.

## References

[1] http://www.healthypeople.gov/2020/topics-objectives/topic/lesbian-gay-bisexual-and-transgender-health/objectives. Accessed Aug 2016.

[2] https://iom.nationalacademies.org/Reports/2011/The-Health-of-Lesbian-Gay-Bisexual-and-Transgender-People.aspx. Accessed Aug 2016.

[3] Rosario M, Corliss HL, Everett BG, Reisner SL, Austin SB, Buchting FO (2014). Sexual orientation disparities in cancer-related risk behaviors of tobacco, alcohol, sexual behaviors, and diet and physical activity: pooled Youth Risk Behavior Surveys. Am J Public Health.

[4] Operario D, Gamarel KE, Grin BM, Lee JH, Kahler CW, Marshall BD (2015). Sexual Minority Health Disparities in Adult Men and Women in the United States: National Health and Nutrition Examination Survey, 2001–2010. Am J Public Health.

[5] Ward BW, Dahlhamer JM, Galinsky AM, Joestl SS. Sexual orientation and health among U.S. adults: national health interview survey, 2013. Natl Health Stat Report 2014, (77):1–10 25025690

[6] Dahlhamer JM, Galinsky AM, Joestl SS, Ward BW. Sexual orientation in the 2013 national health interview survey: a quality assessment. Vital Health Stat 2 2014, (169):1–32 25510624

[7] Diamant AL, Wold C, Spritzer K, Gelberg L (2000). Health behaviors, health status, and access to and use of health care: a population-based study of lesbian, bisexual, and heterosexual women. Arch Fam Med.

[8] Hatzenbuehler ML, Bellatorre A, Lee Y, Finch BK, Muennig P, Fiscella K (2014). Structural stigma and all-cause mortality in sexual minority populations. Soc Sci Med.

[9] Buchmueller T, Carpenter CS (2010). Disparities in health insurance coverage, access, and outcomes for individuals in same-sex versus different-sex relationships, 2000–2007. Am J Public Health.

[10] http://www.cdc.gov/nchs/data/nhsr/nhsr077.pdf. Accessed Aug 2016.

[11] Meyer IH (1995). Minority stress and mental health in gay men. J Health Soc Behav.

[12] Hatzenbuehler ML, McLaughlin KA, Keyes KM, Hasin DS (2010). The impact of institutional discrimination on psychiatric disorders in lesbian, gay, and bisexual populations: a prospective study. Am J Public Health.

[13] Ponce NA, Cochran SD, Pizer JC, Mays VM (2010). The effects of unequal access to health insurance for same-sex couples in California. Health Aff (Millwood).

[14] Bauermeister JA, Meanley S, Hickok A, Pingel E, Vanhemert W, Loveluck J (2014). Sexuality-related work discrimination and its association with the health of sexual minority emerging and young adult men in the Detroit Metro Area. Sex Res Social Policy.

[15] Hatzenbuehler ML (2009). How does sexual minority stigma “get under the skin”? A psychological mediation framework. Psychol Bull.

[16] http://www.cdc/gov/pub/Health_Statistics/NCHS/Dataset_Documentation/NHIS/2007/srvydesc.pdf. Accessed Aug 2016

[17] Office of Disease Prevention and Health Promotion. Dietary guidelines. Available at: http://www.health.gov/dietary guidelines. Accessed Aug 2016

[18] Alvarez GG, Ayas NT (2004). The impact of daily sleep duration on health: a review of the literature. Prog Cardiovasc Nurs.

[19] Grandner MA, Hale L, Moore M, Patel NP (2010). Mortality associated with short sleep duration: The evidence, the possible mechanisms, and the future. Sleep Med Rev.

[20] Barros AJ, Hirakata VN (2003). Alternatives for logistic regression in cross-sectional studies: an empirical comparison of models that directly estimate the prevalence ratio. BMC Med Res Methodol.

[21] Duryea DG, Frantz TT (2011). An examination of drinkers’ consequences by sexual orientation. J Am Coll Health.

[22] Valanis BG, Bowen DJ, Bassford T, Whitlock E, Charney P, Carter RA (2000). Sexual orientation and health: comparisons in the women’s health initiative sample. Arch Fam Med.

[23] Cochran SD, Keenan C, Schober C, Mays VM (2000). Estimates of alcohol use and clinical treatment needs among homosexually active men and women in the U.S. population. J Consult Clin Psychol.

[24] Burgard SA, Cochran SD, Mays VM (2005). Alcohol and tobacco use patterns among heterosexually and homosexually experienced California women. Drug Alcohol Depend.

[25] Hughes TL (2003). Lesbians’ drinking patterns: beyond the data. Subst Use Misuse.

[26] Marshall BD, Shoveller JA, Kahler CW, Koblin BA, Mayer KH, Mimiaga MJ (2015). Heavy drinking trajectories among men who have sex with men: a longitudinal, group-based analysis. Alcohol Clin Exp Res.

[27] Lewis NM (2009). Mental health in sexual minorities: recent indicators, trends, and their relationships to place in North America and Europe. Health Place.

[28] Cochran SD, Mays VM, Sullivan JG (2003). Prevalence of mental disorders, psychological distress, and mental health services use among lesbian, gay, and bisexual adults in the United States. J Consult Clin Psychol.

[29] Mason TB, Lewis RJ (2015). Minority stress and binge eating among lesbian and bisexual women. J Homosex.

[30] Agenor M, Peitzmeier S, Gordon AR, Haneuse S, Potter JE, Austin SB (2015). Sexual Orientation Identity Disparities in Awareness and Initiation of the Human Papillomavirus Vaccine Among U.S. Women and Girls: A National Survey. Ann Intern Med.

[31] Gonzales G, Blewett LA (2014). National and state-specific health insurance disparities for adults in same-sex relationships. Am J Public Health.

[32] Heck JE, Sell RL, Gorin SS (2006). Health care access among individuals involved in same-sex relationships. Am J Public Health.

[33] Rahman Q, Silber K (2000). Sexual orientation and the sleep-wake cycle: a preliminary investigation. Arch Sex Behav.

[34] Meyer IH (2003). Prejudice, social stress, and mental health in lesbian, gay, and bisexual populations: conceptual issues and research evidence. Psychol Bull.

[35] Eckleberry-Hunt J, Dohrenwend A (2005). Sociocultural interpretations of social phobia in a non-heterosexual female. J Homosex.

[36] Mays VM, Cochran SD (2001). Mental health correlates of perceived discrimination among lesbian, gay, and bisexual adults in the United States. Am J Public Health.

[37] Herek GM (2009). Hate crimes and stigma-related experiences among sexual minority adults in the United States: prevalence estimates from a national probability sample. J Interpers Violence.

[38] Diamond, Lisa M. A New View of Lesbian Subtypes: Stable Versus Fluid Identity Trajectories Over an 8**-**year Peruid. Psychology of Women Quarterly 29.2 (2005): 119–128.

[39] Katz-Wise SL, Hyde JS (2015). Sexual fluidity and related attitudes and beliefs among young adults with a same-gender orientation. Arch Sex Behav.

[40] Coulter RW, Kenst KS, Bowen DJ, Scout (2014). Research funded by the National Institutes of Health on the health of lesbian, gay, bisexual, and transgender populations. Am J Public Health.

[41] Everett BG, Rosario M, McLaughlin KA, Austin SB (2014). Sexual orientation and gender differences in markers of inflammation and immune functioning. Ann Behav Med.

[42] Bowleg L (2012). The problem with the phrase women and minorities: intersectionality-an important theoretical framework for public health. Am J Public Health.

